# Formability of Ultrasonically Additive Manufactured Ti-Al Thin Foil Laminates

**DOI:** 10.3390/ma12203402

**Published:** 2019-10-17

**Authors:** İrfan Kaya, Ömer Necati Cora, Muammer Koç

**Affiliations:** 1 Department of Mechanical Engineering, Faculty of Engineering, Eskisehir Technical University, Eskisehir 26555, Turkey; 2 Department of Mechanical Engineering, Karadeniz Technical University, Trabzon 61080, Turkey; oncora@ktu.edu.tr; 3 Division of Sustainable Development (DSD), College of Science & Engineering (CSE), Hamad bin Khalifa University (HBKU), Qatar Foundation (QF), Education City, Doha 5825, Qatar

**Keywords:** Ti-Al composites, ultrasonic consolidation, laminated metal composites, warm hydroforming, formability, bulge test

## Abstract

This study investigates the effect of strain rates and temperatures on the mechanical behavior of ultrasonically consolidated Titanium–Aluminum thin foils to understand and characterize their formability. To this goal, laminated composite samples with a distinct number of layers were bonded using ultrasonic consolidation. Then, tensile and biaxial hydraulic bulge tests at different strain rates and temperature conditions were conducted. The effect of the sample orientation on the mechanical response was also examined. Tensile and hydraulic bulge tests results were compared to observe differences in ultimate tensile strength and strain levels under uniaxial and biaxial loading conditions. The effects of loading condition, strain rate, and temperature on the material response were analyzed and discussed on the basis of test results. In general, it was concluded that the maximum elongation values attained were higher for the samples subtracted along the sonotrode movement direction compared to those obtained from the normal to sonotrode movement direction. The elongation was obtained as high as 46% for seven bi-layered samples at high-temperature ranges of 200–300 °C. Hydraulic bulge test results showed that elongation improved as the number of bi-layers increased, yet the ultimate strength values did not change significantly indicating an expansion of the formability window.

## 1. Introduction

In recent years, laminated metal composites (LMCs) that combine the superior features of different metals have gained considerable attention as they improves properties such as high strength, stiffness, and toughness [1,2,3,4,5]. In particular, the titanium–titanium tri-aluminide (Ti-Al3Ti) composite system is a great candidate for structural applications due to the combination of high strength, stiffness, and toughness at a lower density of monolithic titanium. In addition, the Ti-Al3Ti system is more economical than monolithic titanium because Al is relatively inexpensive compared to Ti [6]. Similarly, lightweight Ti-Al LMCs have been increasingly exploited in various applications including defense, aerospace, and automotive [6]. For example, LMCs that consist of commercial pure Ti (CP-1) and pure Al (AA1100) layers have been used in armor applications such as in mine blast mitigation as well as in civil aviation [7].

Wu et al. fabricated and designed a brittle/ductile Ti-Al laminated composite using reaction annealing of pure Ti foil and Al foil to investigate their mechanical behavior [8]. They noted that the combination of good strength and ductility was obtained. 

Additive manufacturing (AM), also dubbed as additive fabrication, additive processes, additive techniques, additive layer manufacturing, and layer manufacturing, is a recent production method to fabricate 3D parts by adding materials layer by layer [9]. In recent years, AM has attracted much attention due to its significant advantages such as design freedom, tool and die cost elimination, reducing number of subparts, and short lead times compared to conventional manufacturing methods [10]. As a result, AM is vitally important technology where the repeatability and accuracy of properties are the utmost concerns.

Commercial metal laminates have been fabricated by means of a variety of techniques. The most common of which are adhesive bonding, co-extrusion, ultrasonic consolidation, and brazing. Among those, ultrasonic consolidation enables welding of many commercially available metals such as aluminum, titanium, magnesium, copper, and steel [11,12]. 

**Figure 1:**
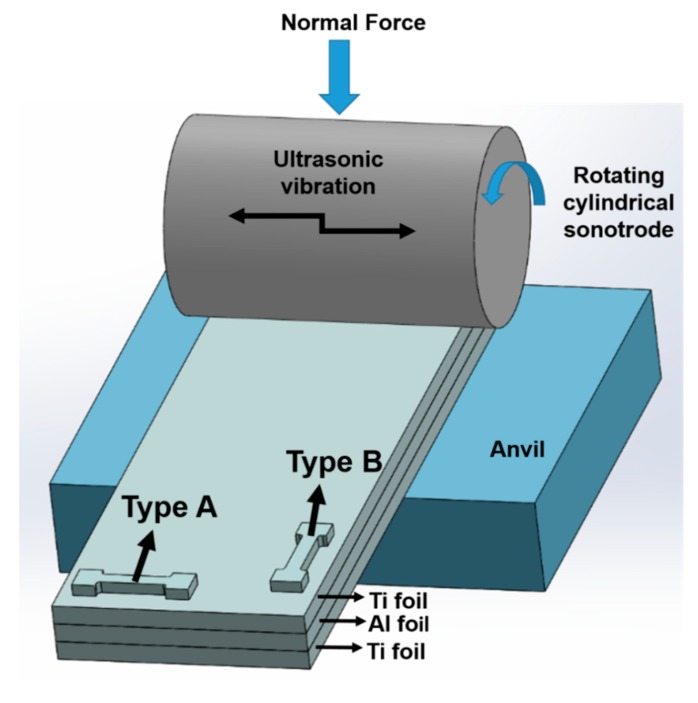
Schematic illustration of UC and test sample extractions.

Ultrasonic consolidation (UC) is an ultrasonic additive manufacturing process (Figure 1). It is a solid-state bonding method where thin similar or dissimilar metal foils are ultrasonically welded one above the other and processed periodically to form a final piece [13,14,15,16,17]. The UC process shortens the production time and production costs of parts while at the same time it offers to weld a very wide range of metals [13].

In the UC process, as illustrated in Figure 1, a rotating ultrasonic sonotrode is brought into contact with the metallic foil to be joined under controlled pressure and normal load. The foil is then combined with ultrasonic vibrations into pre-separated metal at the bottom [12,18,19,20,21,22]. High-frequency ultrasonic vibration creates friction that allows two metals to weld together [23]. The oxides and contaminants on the contacting surface are eliminated by high friction. As a result, plastic flow and clean surface area which allow metal-to-metal contact for bonding are obtained [24,25]. Mechanical interlocking between metals also takes place at a relatively lower temperature than other methods [26,27].

The literature is scarce in terms of mechanical properties of ultrasonically additive manufactured laminated metal composites that consist of pure Ti (CP-1) and pure Al (AA1100) foils. Most of the existing literature on LMC of these alloys have been focused on the tensile properties at the room temperature. Yet, the effect of the temperature on the mechanical properties has not been considered, even though this can be a critical design factor in alloys exposed to relatively high temperatures. The motivation in the current study, is, to reveal the deformation behavior of such LMCs under complex and uniaxial and bi-axial loading conditions. This study could shed light for further understanding of the forming limits of ultrasonic additive manufactured Ti-Al LMCs. Another aim of the study is to reveal the formability properties of Ti-Al LMCs for final product use purposes.

Early work by the authors focused on Ti-Al LMCs that were ultrasonically welded on a 1.524-mm-thick aluminum substrate, which resulted in a different number of bi-layered (one, three and five bi-layered) LMC configurations [28]. In the current study, the emphasis is placed on Ti-Al LMCs without the thick aluminum substrate in three, five and seven bi-layered LMC configurations. Each bi-layer consists of one Al and one Ti layer, each 127 µm in thickness. In order to investigate the deformation characteristics of ultrasonically consolidated LMCs, both tensile and bulge tests were conducted at four different temperatures of 25 °C, 100 °C, 200 °C, and 300 °C, and under the strain rates of 0.013/s, and 0.0013/s. 

## 2. Materials and Methods 

The foils that are used to build up the laminates in the current study were commercial pure Ti and pure Al 1100, each with a thickness of 127 µm. Ultrasonic consolidation was employed to fabricate bi-layered Ti-Al laminated metal composites in three different configurations. The lowermost and uppermost layers were Ti, and during the consolidation process, the sonotrode passed on Ti layers. All the samples were consolidated using a sonotrode frequency of 20 kHz under certain normal force. Each bi-layer (Ti on top, Al at bottom) was welded together onto the previous layer except for the first consolidation in which two bi-layers were welded together as the first step. The overall thickness of three, five and seven bi-layered samples were 0.889, 1.397 and 1.905 mm, respectively. The overall Al contents (in weight %) for the bi-layered samples were obtained as 31%, 33.3%, and 34.4% for three, five, and seven bi-layered samples, respectively. Upon completing the consolidation of the required number of bilayers, the parts were heat treated for 4 h at 480 °C to improve metallurgical bonding.

**Figure 2:**
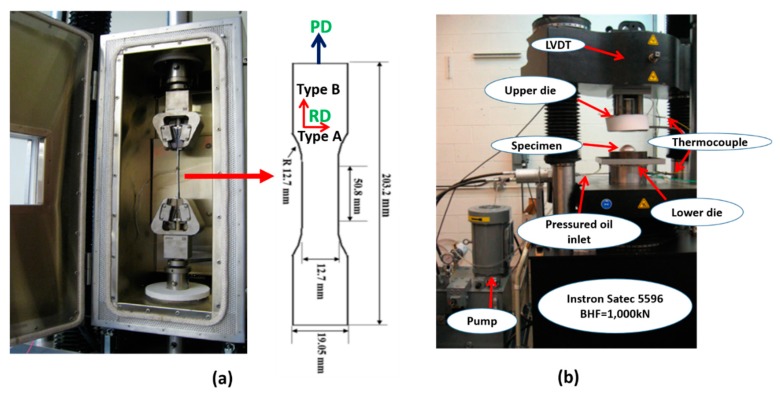
(**a**) Warm tensile test device and tensile test specimen dimensions and configurations, (**b**) hydraulic bulge testing device.

Dog bone-shaped tensile specimens were extracted from LMCs blanks in two different orientations and named as Types A and B. Type A refers to the sample extracted from along the sonotrode vibration direction (normal to the travel of the sonotrode) while Type B samples were acquired along the direction of the travel of the sonotrode (Figure 1). Ti-Al LMCs tensile test samples were obtained by means of abrasive water jet machining according to the ASTM E8-04 standard (Figure 2a). Tensile coupons for thin titanium samples are named as Type A if the rolling direction is perpendicular to the pulling direction (PD in Figure 2a), while it is named as Type B if the rolling direction is parallel to the pulling direction. Uniaxial tensile tests were performed on a 10 kN electromechanical MTS machine equipped with a furnace with strain rates of 0.013/s and 0.0013/s, at four different temperatures (25 °C, 100 °C, 200 °C, and 300 °C). The K-type thermocouple was employed to measure the temperature of the test samples from the mid-section. After the specimens reached the desired test temperature, they were pulled with a constant cross-head speed until they broke.

The flow (stress-strain) curves were determined by the warm hydraulic bulge test machine shown in Figure 2b. Omega PX05 pressure transducer (Omega Engineering Inc., Norwalk, CT, USA) was utilized to obtain the pressure given to the system during bulging. The height of the dome was measured by a linear variable differential transformer (LVDT) (Omega Engineering Inc., Norwalk, CT, USA). The detail regarding the experimental setup can be found elsewhere in the literature [28].

Each bulge and tensile test were conducted three times, and the average of three tests was reported as the final result. 

Upon bulge tests, samples were first cut by a high-precision electrical discharge machine (EDM). The samples surfaces were polished for the optical microscopy and microhardness tests. The microhardness tests were conducted with 100 g.f. (981.2 mN) force by using Struers model Duramin 5 microhardness tester. Ten measurements were performed at each layer of Al and Ti, and an average of these was noted as a final hardness value.

## 3. Results and Discussion

**Figure 3:**
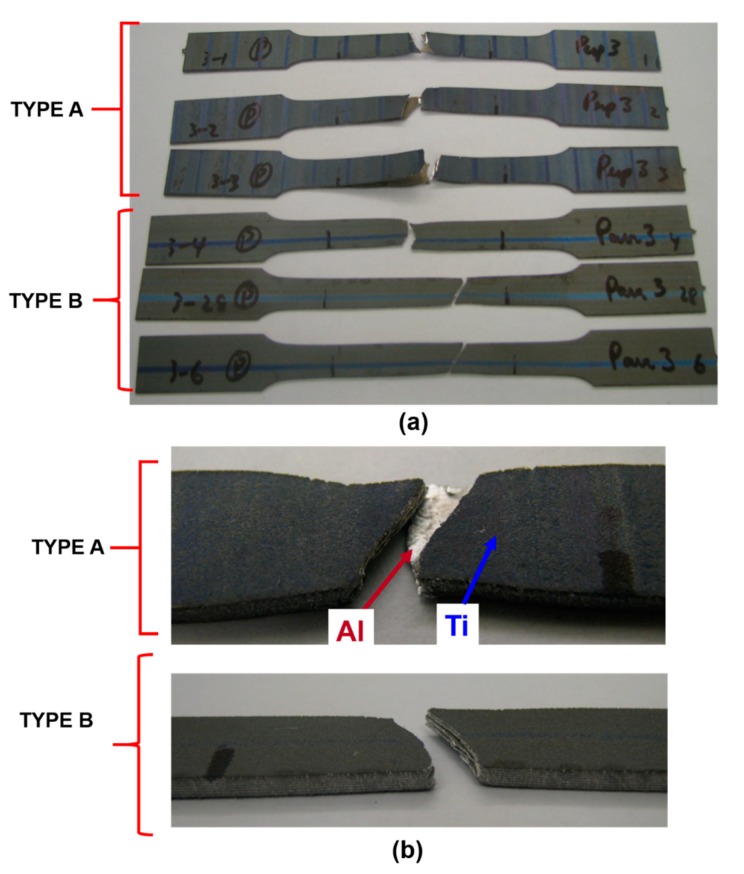
Different failure types observed after tensile tests performed at a strain rate of 0.0013/s at RT for (**a**) three bi-layered and (**b**) seven bi-layered samples.

Figure 3a shows three bi-layered tensile samples that were tested at a strain rate of 0.0013/s, and at room temperature (RT). As it can be seen from Figure 3a, different failure patterns appeared for both Type A and Type B samples in three bi-layered configurations. Increased delamination tendency was observed for Type A three bilayered samples at RT (25 °C) tests. More ductile and uniform elongation/ fracture (with 45° angle to pulling direction) was also noted for both Ti, Al layers among Type B samples when compared with Type A samples (Figure 3b). This can be attributed to the increasing grain orientation when both rolling and pulling directions coincide. These results are in agreement with the literature [29,30]. 

**Figure 4:**
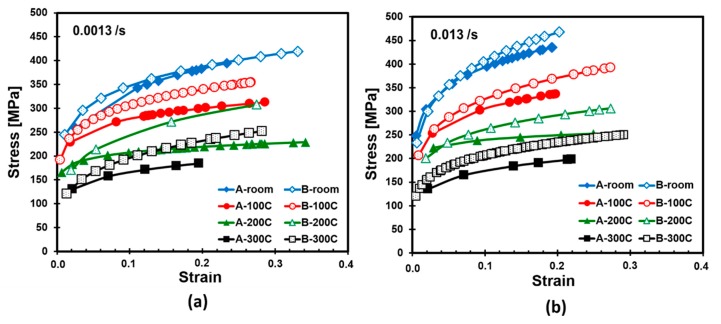
Stress-strain curves based on tensile tests for thin titanium foil at (**a**) 0.0013/s and (**b**) 0.013/s loading strain rates.

In order to assess the effect of ultrasonic consolidation on the mechanical properties, not only the laminated but also individual foils were subjected to uniaxial tensile tests. Figure 4, for example, illustrates stress-strain curves of titanium sheets 0.127 mm in thickness. The tensile tests were conducted at strain rates of 0.0013/s and 0.013/s. It was observed that the strength decreases with increasing test temperature for both Types A and B titanium samples, as expected. The highest strain value of 0.33 was obtained under 420 MPa stress level at room temperature and at a 0.0013/s strain rate for Type B sample. Type B samples exhibited higher strength compared to Type A samples in all cases. When Type A samples are examined in Figure 4a,b, it can be seen that the higher strain values were obtained at a strain rate of 0.0013/s, in general. The strain values of 0.19, 0.20, 0.25, and 0.22 were recorded at test temperatures of 25 °C, 100 °C, 200 °C, and 300 °C, respectively, at 0.013/s strain rate while corresponding strain values were recorded as 0.21, 0.29, 0.35, and 0.20 at a strain rate of 0.0013/s.

**Figure 5:**
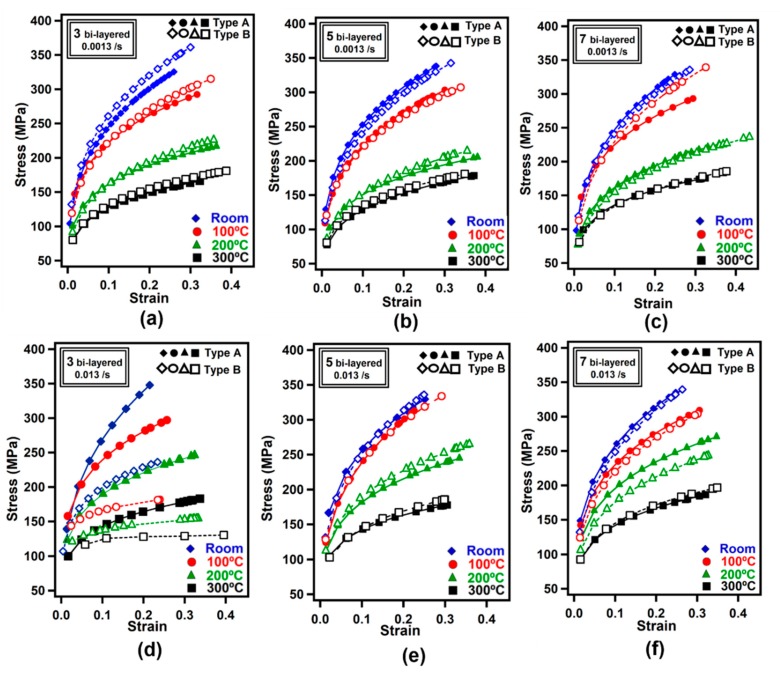
Stress-strain curves obtained for laminated metal composites obtained from tensile tests; (**a**–**c**) at a strain rate of 0.0013/s, and (**d**–**f**) at a strain rate of 0.013/s.

Figure 5 demonstrates the stress-strain curves of laminated metal composites. Figure 5a–c shows the tensile test results under 0.0013/s strain rate while Figure 5d–f shows the results under 0.013/s strain rate. 

In Figure 5a–c, the strength level did not change significantly no matter the bilayer number and test temperature. The effect of sample orientation on the results diminished as the number of bilayers increased, as can be noticed from Figure 5a–c. In order to increase the number of layers, it is necessary to apply an increased number of ultrasonic treatments to the sample. It is therefore assumed that grain orientation effect diminishes between Types A and B samples due to increased bonding between individual foils. For the LMCs, the development of interfacial bonding ability has a significant effect on the mechanical properties [31]. The highest strains at room temperature were recorded as 0.22 and 0.29 for Types A and B samples, respectively. The ultimate tensile stresses decreased with increasing temperature in all cases as shown in Figure 5. As can be noticed from Figure 5e, test temperature increase from room temperature to 300 °C decreased the ultimate stress from 325 MPa to 177 MPa for Type A samples. It is widely known that increasing the temperature results in increased distance between the atoms which decreases the necessary force to deform the materials so the ultimate tensile strength.

**Figure 6:**
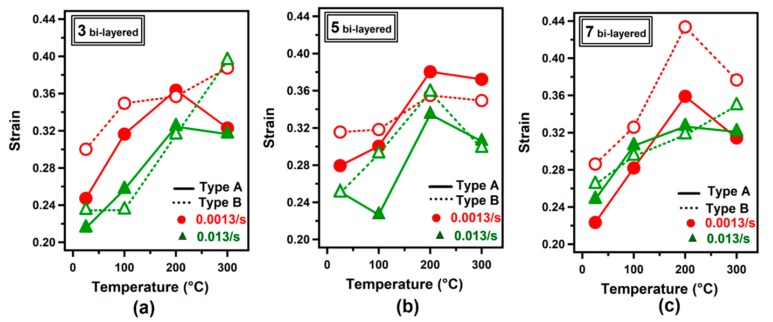
Strain-temperature variation based on tensile tests: (**a**) three bi-layered, (**b**) five bi-layered and (**c**) seven bi-layered composites.

Figure 6a–c shows the strain values obtained for three, five, and seven bi-layered LMCs as a function of test temperature. LMCs were deformed in uniaxial tensile mode at a strain rate of 0.0013/s and 0.013/s.

In general, slight strain values are higher for Type B samples compared to those obtained for Type A samples. The maximum strain values for three, five and seven bi-layered samples at 100 °C were recorded as 0.32, 0.30 and 0.28, respectively for Type A samples at a strain rate of 0.0013/s. At the same test condition, the strain values of 0.35, 0.32 and 0.33 were acquired respectively for Type B samples.

The highest strain value for three bi-layered samples was obtained as 0.39 at a strain rate of 0.013/s and test temperature of 300 °C for Type B configuration. At room temperature, on the other hand, the maximum strain value of 0.30 was obtained at a strain rate of 0.0013/s for the three bi-layered Type B sample (Figure 6a).

In most cases, elevation in test temperature up to 200 °C yielded elevated elongation. As an example, the strain was recorded as 0.23 at 100 °C while it was obtained as 0.33 at 200 °C at a strain rate of 0.013/s for the five bi-layered Type A sample (Figure 6b). The highest strain value was obtained as 0.43 at a strain rate of 0.0013/s and at a test temperature of 200 °C for the seven bi-layered Type B sample.

**Figure 7:**
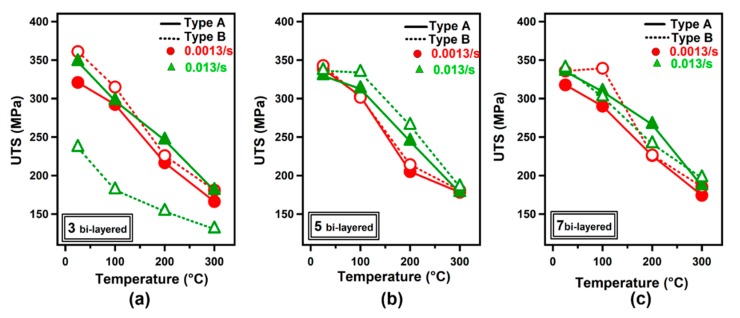
Variation of ultimate tensile strength values with respect to the tensile test temperature for (**a**) three bi-layered, (**b**) five bi-layered and (**c**) seven bi-layered LMCs.

Figure 7a–c illustrates the ultimate tensile strength (UTS) of three, five and seven bi-layered samples at various test temperatures obtained from tensile tests at selected strain rates. One can conclude by examining Figure 7a–c that an increased number of bi-layers did not affect the ultimate tensile strength considerably at all strain rates and in both sample orientations. At a strain rate of 0.013/s, the ultimate tensile strength values were noted as 335 MPa and 186 MPa at 25 °C and 300 °C test temperatures, respectively, for the seven bi-layered Type A sample while corresponding values for the Type A three bi-layered sample were 347 MPa and 181 MPa.

In addition to the effect of temperature on the strength level of LMCs, the strain rate dependence of stress-strain curves was also investigated. As shown in Figure 7a–c, the strain rate did not influence stress levels significantly for three and five bi-layered Type A samples. For example, ultimate tensile strength values were recorded as 312 MPa and 303 MPa at strain rates of 0.013/s and 0.0013/s, respectively, at 100 °C for the Type A sample with five bi-layers. The ultimate tensile strength values were obtained as 334 MPa and 302 MPa at a strain rate of 0.013/s and 0.0013/s, respectively, at 100 °C for the Type B sample with five bi-layers.

**Figure 8:**
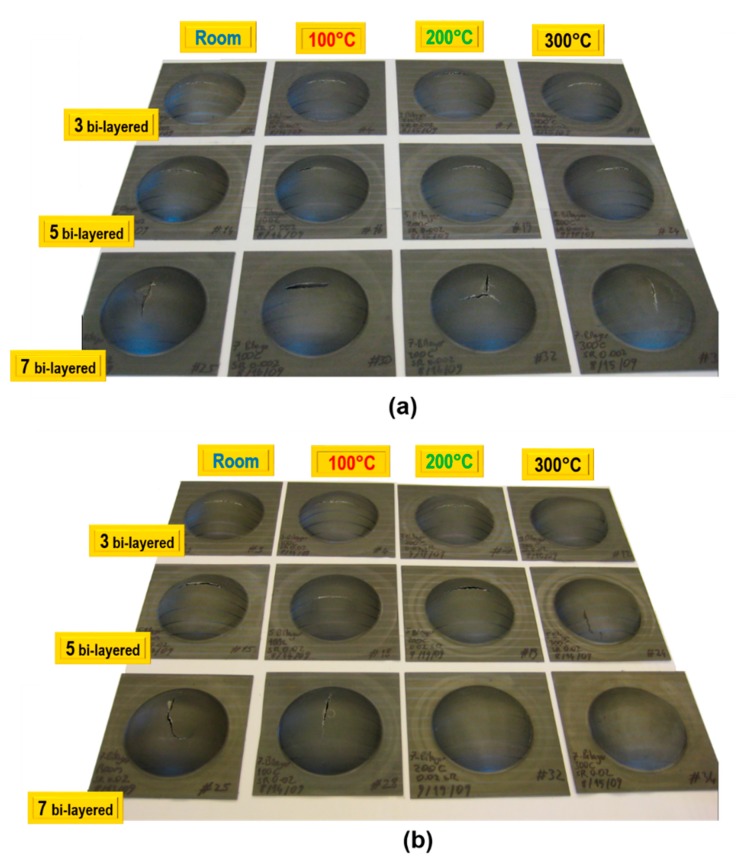
LMC samples in a different number of bi-layers tested at (**a**) 0.0013/s and (**b**) 0.013/s strain rates.

Characterization of LMC samples was also carried out by means of bulge test analyses. LMC blanks were bulged with hydraulic fluid till they ruptured. Failure track was observed to be parallel to the rolling direction for three and five bi-layered samples, yet it varied for seven bi-layered blanks. Besides, the fracture location was observed at the center of the dome for all the bulged specimens at a strain rate of 0.0013/s as shown in Figure 8a. In Figure 8b, on the other hand, the fracture location was observed at the center of the dome for most cases.

**Figure 9:**
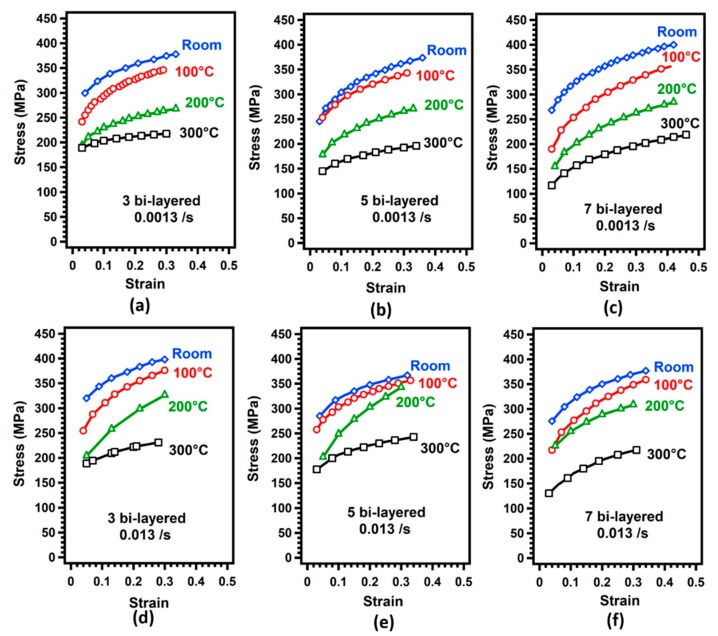
Stress-strain curves obtained from bulge tests for laminated metal composites: (**a**–**c**) at a strain rate of 0.0013/s and (**b**–**f**) at a strain rate of 0.013/s.

Figure 9 shows the flow curves of deformed LMCs at different strain rates obtained from bulge tests at various temperatures. Figure 9a–c, in particular, illustrate the bulge test results acquired at 0.0013/s strain rate while Figure 9d–c shows the results obtained at a strain rate of 0.013/s.

As expected and experienced in tensile test results, an increase in test temperature resulted in a decrease in strength values, yet it did not affect the elongation for the same number of bi-layered samples at both strain rates. It is expected to see (for most metals) a certain increase in elongation with increasing temperature. Nevertheless, the material of interest in this study (Ti-Al LMC) is not a uniform material. Both the coefficient of thermal expansion and formability are quite different for Ti and Al, so their responses to temperature change. Early failure of one of those components affected the overall response of the LMC, however; temperature increase resulted in the same effect (decrease in strength) for both components. On the other hand, it was observed that an increasing number of bi-layers improved the elongation, yet it did not change the ultimate strength values. This is more pronounced at a strain rate of 0.0013/s and it can be attributed to the stronger bonds (increased number of sonotrode passes in building up samples) for five and seven bi-layered samples.

**Figure 10:**
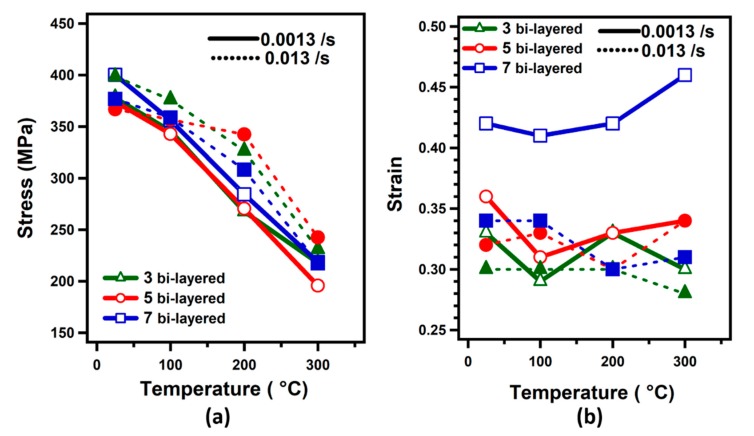
(**a**) Stress-temperature and (**b**) strain-temperature curves for Ti-Al laminated metal composites obtained from bulge tests at different strain rates.

Figure 10a illustrates the ultimate strength of three, five and seven bi-layered samples obtained at 25 °C, 100 °C, 200 °C, and 300 °C for a strain rate of 0.0013/s and 0.013/s. The maximum stress level of 400 MPa was obtained at a strain rate of 0.0013/s at 25 °C for seven bi-layered samples. It was again noted that the number of bi-layers does not seem to affect the stress level at selected test temperatures and at both strain rates. At a strain rate of 0.0013/s, and 100 °C, the maximum stress values were obtained as 346 MPa, 343 MPa, and 356 MPa, for three bi-layered, five bi-layered and seven bi-layered samples, respectively. Similarly, the maximum stress values at a faster strain rate (0.013/s) were noted as 231 MPa, 243 MPa, and 217 MPa for three bi-layered, five bi-layered and seven bi-layered samples, respectively, at 300 °C. For the bulge tests performed at 200 °C, it was noticed that the ultimate strength varied according to the strain rate. When the strain rate of 0.013/s and temperature of 200 °C were adopted for three bi-layered specimens, the maximum stress value was obtained as 326 MPa, whereas when the 0.0013/s strain rate was applied, the maximum stress value was 267 MPa. For five bi-layered samples, the maximum stress values of 342 MPa and 279 MPa were obtained at 0.013/s and 0.0013/s strain rates, respectively.

Figure 10b shows the strain of three, five and seven bi-layered samples as a function of the test temperature at selected strain rates. Examining the results in Figure 10b, within the same bi-layered materials, seven bi-layered samples showed the highest elongation at a slow strain rate (0.0013/s). The highest strain of 0.46 was obtained at 300 °C temperature.

**Figure 11:**
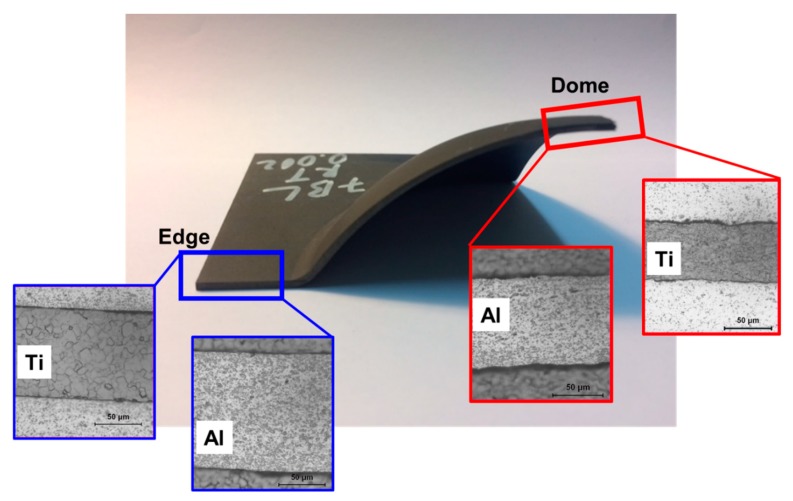
The images showing the Al and Ti layers at the edge and dome regions of the bulged sample.

Even though the LMCs were built from 127-µm-thick foils, this thickness is reduced over the bulged part’s different zones. Figure 11 shows the two critical regions of the seven bi-layered bulged sample. The edge region of the LMC part is exposed to a blank holder force where relatively less thickness change is expected. The dome region, contrarily, experiences the maximum thinning. As seen in Figure 11, the thickness of Al and Ti layers at the dome are clearly smaller than those measured at the edge region. Upon bulging at room temperature, the mean Ti and Al thickness were 97 µm and 127 µm at the edge region, respectively, which were recorded as 62 µm and 88 µm, respectively, at the dome region, in which there was a greater extent of thinning, so the deformation occurred. The thickness value for foils significantly decreased through deformation at 300 °C. For example, the mean Ti and Al thicknesses were measured as 68 µm and 62 µm, respectively, at the edge region while mean thickness values of Ti and Al layers at the dome region were noted as 43 µm and 39 µm, respectively. Although the Ti yield strength of Ti is higher than that for Al, the Ti layers’ thicknesses were decreased more than those for Al.

**Figure 12:**
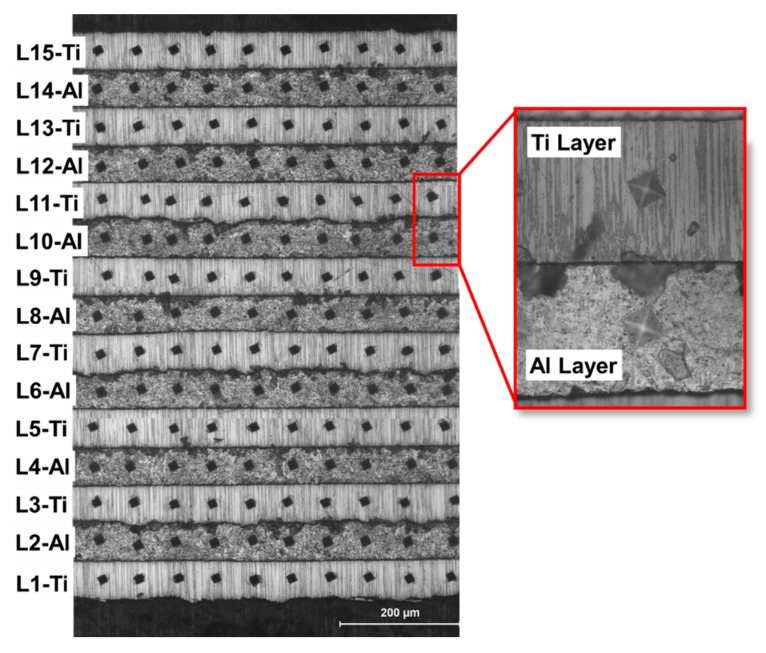
Micro-indentations on different layers of bulged sample at RT at 0.0013/s strain rate of Ti-Al laminated composite.

Microhardness measurements were also performed to comprehend the integral influence of hydroforming and UC on the hardness of individual layers. Ten different measurements on each layer were obtained on seven bi-layered Ti-Al LMC which was deformed at 0.0013/s strain rate, and at two different temperatures, namely room temperature and 300 °C. Figure 12 presents Micro-Vickers hardness measurements obtained from each layer through Ti-Al LMC’s thickness.

**Figure 13:**
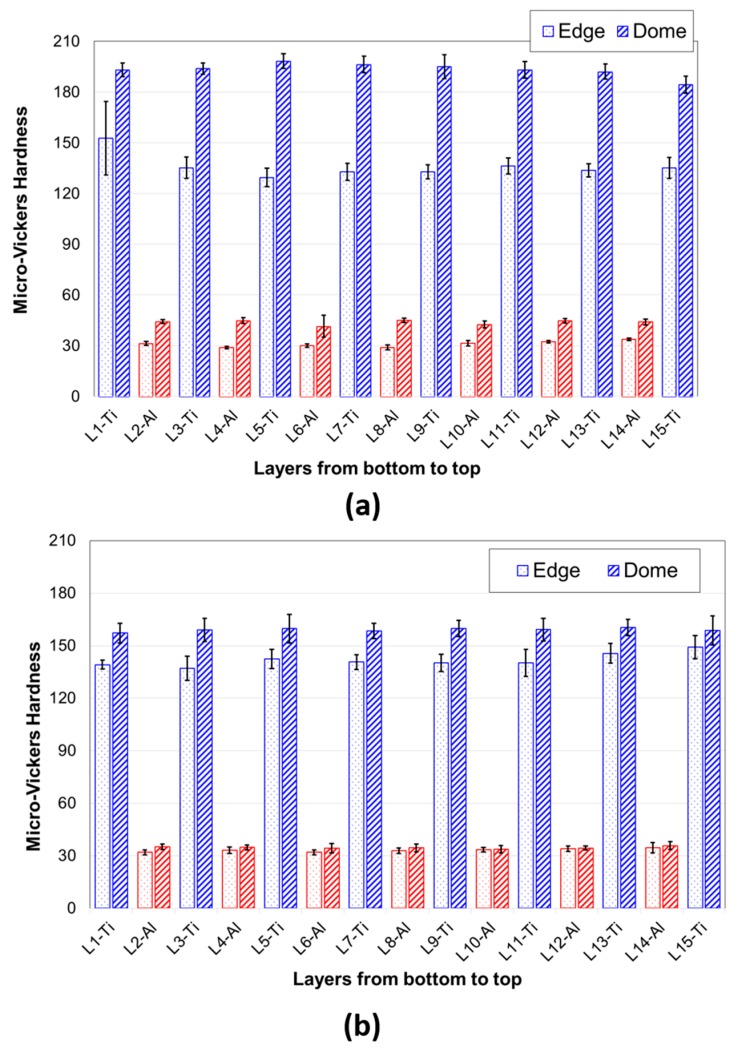
Micro-Vickers hardness values obtained at individual foils on edge and dome regions (**a**) for a bulged sample at RT and (**b**) for a bulged sample at 300 °C.

The hardness values of the bulged sample at RT are shown in Figure 13a while the hardness values of the bulged sample at 300 °C are presented in Figure 13b. In Figure 13a, it is clear that the hardness values at the dome region are higher than those obtained at the edge region. This is attributed to the fact that edge region is subjected negligible deformation while the dome experiences the maximum deformation, so the work-hardening. The mean hardness values for Ti foils at the edge and dome were 138 and 195, respectively, for the bulged sample at RT, whereas those values for Al foils at the edge and dome were 30 and 44, respectively. The mean Ti foils hardness values at the edge and dome were 142 and 159, respectively, for the bulged sample at 300 °C, while corresponding values for Al foils at the edge and dome were noted as 33 and 35, respectively. It is seen that hardness values of Al foils were significantly smaller and showed variations to a smaller extent over the hardness variations of Ti foils. As it can be seen from Figure 13b, the hardness values of both Ti and Al foils decreased with increasing forming temperature. One can conclude that the high temperature forming at 300 °C had an important role in deformation behavior, unlike the deformation behavior of the bulged sample at RT.

In the previous study by authors [28], the ultrasonic additive manufacturing of LMC blanks was carried out on a thick aluminum substrate and consisted of a number of bi-layers. In the current study, on the other hand, LMC blanks without the thick aluminum substrate were tested at the same temperature levels and strain rates.

**Figure 14:**
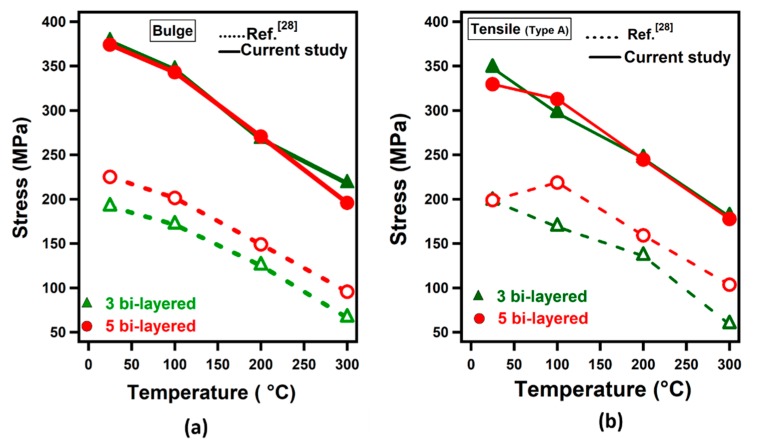
Failure stress-temperature curves: (**a**) Bulge test results at a strain rate of 0.0013/s and (**b**) tensile test results at a strain rate of 0.013/s and 0.017/s of the current study and previous study by authors [28].

Figure 14a,b shows bulge and tensile test results, respectively, of the current study and previous study. Figure 14a displays a variation of ultimate tensile strengths as a function of test temperature in bulge tests. It is observed that the LMCs have higher strength than the previous study. In the current study, the ultimate strength value is almost the same for three bi-layered and five bi-layered samples for the same test conditions. Nevertheless, strength value increases with an increasing number of bi-layers mainly due to increasing Ti content in LMCs. The strength value of 192 MPa was obtained for three bi-layered sample at 25 °C while it was acquired as 225 MPa for five bi-layered samples tested at 25 °C.

When compared with the results of the previous study, it was observed that the failure stress levels in the current study are significantly higher than those in the previous study (Table 1). This is mainly due to the fact that the bi-layered samples were built onto a thick aluminum substrate and Al contents (% in weight) are significantly different. For example, three-bilayered samples in the current study have 31% Al in weight compared to 75% Al in weight in the previous study [28]. It was concluded that the Al content dictated the failure stress of the laminated metal composites.

It was observed that the strength and elongation of laminated metal composites were affected by the aluminum content. The strength levels of the laminated composites (31–34.4% Al or 65.6–69% Ti in weight) were as high as 80% of Ti in the current study, and 45% of Ti in the previous study carried out by the authors (67–75% Al or 25–33% Ti in weight) in room temperature conditions. The formability of the laminated metal composites, on the other hand, was considerably improved with the addition of Al. Therefore, Ti-Al laminated metal composites have considerable potential in weight saving without compromising part strength.

## 4. Conclusions

In this study, first, ultrasonically additive manufactured Ti-Al laminated metal composites (UC-LMC) were obtained in three different configurations (i.e., three, five and seven bi-layered LMCs). Then, these LMCs were tested in terms of uniaxial and biaxial deformation characteristics at four different temperature levels (25 °C, 100 °C, 200 °C, and 300 °C) and at two different strain rates (0.0013/s and 0.013/s). In summary, the following results were obtained:

- The strength decreases with increasing temperature for thin foils of Ti-Al, as expected.
- In tensile tests, samples extracted from along the sonotrode movement (Type B samples) exhibited slightly higher elongations.
- Ultimate strength values were not affected significantly with the sample orientation.
- The effect of temperature on elongation did not yield to a distinct trend when bulge test results were considered; however, increased elongation was observed with increasing tensile test temperature.
- Though both strain rates were assumed to be in quasi-static range, the higher strain rate (0.013/s) led to higher ultimate strength levels, in general.
- Even though the titanium content was almost the same for different numbers of bi-layered samples, seven bi-layered samples were found to be preferable in terms of both formability and strength. This result was attributed to the fact that the higher the number of bi-layered LMC, the more sound bonding due to the higher number of sonotrode passes in building up the samples.
- Finally, the optimum forming temperature was found to be 200 °C, as the further increase led to an insignificant improvement in formability.

## Figures and Tables

**Table 1:** Stress and Strain Values Obtained from Tensile Tests at 0.013/s of three bi-layered Type A and titanium Type A sample (at RT).

Sample	RT		300 °C	
	Strain(pct)	Stress (MPa)	Strain(pct)	Stress (MPa)
Titanium sheet	0.19	435	0.21	198
3bi-layered–Current study	0.21	374	0.31	180
3bi-layered-Ref. [28]	0.32	197	0.4	59
